# Racial Differences in Psychosocial Resources and Mental and Physical Health Outcomes during Pregnancy: A structural equation modeling approach

**DOI:** 10.21203/rs.3.rs-4617998/v1

**Published:** 2024-07-19

**Authors:** Charlotte V. Farewell, Sarah J. Schmiege, Jenn Leiferman

**Affiliations:** University of Colorado – Anschutz Medical Campus

**Keywords:** perinatal health, racial disparities, psychosocial resources, intergenerational transmission, DOHaD

## Abstract

**Objectives:**

Poor prenatal health is of particular concern among minoritized individuals who may experience adverse social determinants of health contributing to the intergenerational transmission of health disparities. The purpose of this study was to investigate associations between psychosocial resources, and mental and physical health among a prenatal sample, and to explore if these relationships vary by race.

**Methods:**

English-speaking pregnant individuals living in the United States were recruited using Centiment (n=340). Participants completed a 121-item cross-sectional survey. We conducted a single- and multi-group structural equation model to test hypothesized relationships, and then investigated differences by pregnant White individuals versus Black, Indigenous, and People of Color (BIPOC).

**Results:**

Our final single-group model exhibited good model fit (χ2 (43) = 99.07, p<.01, CFI = 0.97, SRMR = 0.04, and RMSEA = 0. 06 (0.05 – 0.08)). After controlling for demographic characteristics and social determinants of health, higher levels of mindfulness were statistically significantly related to lower anxiety and depression scores (both p<.01). Higher levels of social supports were statistically significantly related to lower anxiety scores. Scale measurement invariance was confirmed for the multi-group model and the structural model was statistically significantly different between pregnant White individuals and BIPOC in this sample (Δ χ2 (27) = 116.71, p <.01).

**Conclusions:**

Identification of core components of psychosocial resource interventions, consideration of upstream structural determinants, mindfulness and valued-living (MVL)-based strategies, cultural adaptation, and an emphasis on resilience rather than psychopathology may result in improved prenatal health among pregnant individuals traditionally underrepresented in research.

## Introduction

Prenatal mood disorders are increasingly prevalent with approximately 20% of pregnant individuals experiencing depression (Yin et al., 2021) and 22%–40% of pregnant people experiencing anxiety (Grigoriadis et al., 2019). Poor prenatal mental health is associated with a constellation of detrimental pregnancy, birth, and child developmental outcomes (Dadi et al., 2020; Field, 2017; Nicholson et al., 2016). For example, clinical levels of prenatal anxiety are correlated with obstetric complications and poor birth outcomes, such as preeclampsia, premature birth and low birth weight (Abadi-Bavil et al., 2021; Lu et al., 2020; Staneva et al., 2015). Both prenatal depression and anxiety have the potential to negatively affect offspring development and childhood outcomes through the embedding of environmental exposures, as well as compromised bonding between mother and baby (Göbel et al., 2018). The interruption of such bonds may result in delayed cognitive function and socioemotional learning (Bluett-Duncan et al., 2021; Smith et al., 2022), thus perpetuating the intergenerational transmission of poor mental health.

Poor physical health during pregnancy can similarly be deleterious with respect to both maternal and child health outcomes. Approximately 27% of pregnant individuals report experiencing at least one chronic health condition which includes cardiovascular disease, hypertension, cancer, type 2 diabetes, gestational diabetes, overweight/obesity, respiratory diseases (e.g., asthma) and arthritis (Bernell & Howard, 2016; Kersten et al., 2014). Among a representative sample of pregnant individuals in the United States, the most common chronic health conditions were asthma (4.5%), followed by COPD (3.4%), arthritis (3.0%), and heart disease (3.0%) (Chatterjee et al., 2008). Importantly, pregnant individuals with at least one CHC were more likely to deliver by cesarean section and give birth to a premature infant compared to physically healthy pregnant individuals (Kersten et al., 2014). These associations appear to be stronger among low-resourced and minority populations who are often less likely to be insured and have access to prenatal care (Chatterjee et al., 2008).

The comorbidity of poor mental and physical health during pregnancy is of particular concern. The Developmental Origins of Health and Disease (DOHaD) hypothesis suggests that the stressors experienced during the prenatal period are critical for laying the foundations for growth and development outcomes (Ben-Shlomo & Kuh, 2002; Gluckman & Hanson, 2004). Within the DOHaD paradigm, studies are increasingly identifying links between perinatal health and maternal morbidity and disease later in life for offspring. A recent study found significant interactions between depression, high blood pressure, and kidney issues among a prenatal sample and that experiencing these comorbidities increased the risk for poor birth outcomes (Bick et al., 2021; Flynn et al., 2015). This highlights the importance of improving our understanding of modifiable protective factors that can promote prenatal mental and physical health, particularly among low-resourced and minority populations. Individuals who are able to acquire *and* maintain multi-level resources may be better situated to cope with the demands associated with the transition to motherhood (Alvaro et al., 2010; Gallo et al., 2009; Hobfoll, 2002, 2011; Hobfoll et al., 2015; Latendresse, 2009). Psychological (e.g., mindfulness, hope, optimism, self-efficacy, resilience) and social (e.g., support from family members, friends, and significant others) resources may aggregate and interact across the prenatal period to collectively influence prenatal, birth and postpartum outcomes (Cheadle et al., 2020; Dunkel Schetter, 2011; Dunkel Schetter et al., 2013; Hobfoll, 2002).

Mindfulness is one example of a psychological resource that involves the cultivation of moment-to-moment and nonjudgmental awareness of one’s present moment experience (Vieten & Astin, 2008). Numerous studies have found significant associations between mindfulness and prenatal and postpartum (i.e. perinatal) mental health outcomes (Lever Taylor et al., 2016); however, samples are fairly homogenous and often lack inclusion of individuals facing socioeconomic disadvantage and racial and ethnic minority individuals (Burnett-Zeigler et al., 2016; Howard & Khalifeh, 2020; Nillni et al., 2018; Shi & MacBeth, 2017). Hope, optimism, resilience, and self-efficacy are additional, malleable psychological resources, collectively referred to as psychological capital (psycap), which have been found to reduce depression, anxiety, and stress in adults (Luthans & Youssef-Morgan, 2017; Rabenu et al., 2017; Rahimnia et al., 2013; Youssef-Morgan & Luthans, 2015). Though research related to prenatal psycap and mental and physical health outcomes is lacking, past studies have found that greater optimism and self-efficacy are associated with the adoption of healthier coping behaviors, better mental health, and more positive birth outcomes among pregnant individuals (Dunkel Schetter, 2011; Haslam et al., 2006; Yali & Lobel, 2002). Harnessing psycap can strengthen positive interactions with the environment and may be especially critical in shaping stress appraisals to support an adaptive coping process, translating to decreased stress and depression (Lazarus & Folkman, 1987; Rabenu et al., 2017; Song et al., 2019) For example, non-pregnant individuals with higher levels of psycap are more likely to use approach-oriented coping strategies; adoption of these strategies are significantly associated with positive mental and physical health outcomes (Rabenu et al., 2017). Finally, social support may also promote prenatal mental and physical outcomes by buffering the detrimental effects of exposure to adverse life events and social determinants (Tilahune et al., 2022). Specific sources of social supports, including support from family members, friends and significant others, may confer resilience and adaptive coping behaviors thus translating to positive maternal and child health outcomes during these sensitive periods (Milgrom et al., 2019; Razurel et al., 2017).

People of color may experience more demands due to adverse life events but often have fewer resources to respond to these stressors with perpetuates health disparities (Gallo et al., 2009). Providing strategies to enhance these psychological and/or social resources focus on offering support without pathologizing (Hendriks et al., 2019); activities may enhance positive thoughts, promote social supports, and be less stigmatizing compared to traditional psychological supports (Hendriks et al., 2019; Layous et al., 2014), which may be more appealing to minoritized individuals (Hendriks et al., 2019; Ivtzan et al., 2016). Unfortunately, disadvantaged communities are often underrpresented in these intervention studies (Sun et al., 2022). For example, a recent systematic review of 69 mindfulness-based interventions (MBIs) in the U.S. found that among the 45 studies that reported data on race and income, 76% of participants identified as non-Hispanic White and the majority of participants reported an annual household income of greater than $40,000 (Waldron et al., 2018).

Although the root social and economic factors must be addressed to successfully eliminate health disparities, interventions that focus on enhancing multi-level psychosocial resources during early critical periods (e.g., pregnancy) to better cope with stress may be cost-efficient and effective strategies to reduce health inequities. Yet, additional research is needed to analyze linkages between specific psychosocial resources and prenatal mental and physical health outcomes among racially diverse samples to inform the cultural adaptation of psychosocial resource interventions. These data can then be used to ensure fit with the target population and to enhance multi-level resource reservoirs during pregnancy. The purpose of this study was to investigate cross-sectional associations psychosocial resources and mental and physical health outcomes among a prenatal sample, and to explore if these relationships vary by race using a structural equation modeling approach. We hypothesize that the relationships in our final structural model will vary by race suggesting that specific resources may be more protective with respect to prenatal mental and physical health for black, indigenous, and people of color (BIPOC) versus White individuals.

## Methods

### Participants

Table 1 presents demographic characteristics for the final analytical sample, which consisted of English-speaking pregnant individuals living in the United States (n = 340). The sample was recruited using Centiment, an online survey platform that relies on panel recruitment to reach broad and representative audiences. The average age of the participants was 28.46 (SD = 6.34). Approximately two thirds of the participants (69%) were White, 21% were Black, 3% were American Indian or Alaskan Native, 2% were Asian or Pacific Islander, and 8% reported their race as ‘Other’. Eighteen percent (18%) were Hispanic. About one third of the participants were single (33%), 45% were married, and 15% had a domestic partner. Additionally, three quarters (74%) of the sample participants had less than a college degree.

### Procedures

All procedures were approved by Colorado Multiple Institutional Review Board (IRB #: 23-0272). Recruitment was targeted towards social media sites (e.g., Facebook and LinkedIn). To elicit survey participants, Centiment runs batches of notifications to specific subgroups of individuals (based on eligibility criteria) throughout the fielding window of a survey. These notifications include a combination of both email and push notifications based on participant preferences. Participants only see the estimated length of the survey and the reward that they stand to earn before reviewing the survey content. No other information regarding the survey, its subject matter, or how to qualify for the survey is provided in order to avoid selection bias. Individuals are compensated via PayPal accounts. The study consent and survey were administered via Research Electronic Data Capture (REDCap)(Harris et al., 2009). REDCap is a secure, web-based application designed to support data capture for research studies. Participants were recruited over a 2-week period in December of 2022.

### Measures

Participants completed a 121-item survey which consisted of questions related to demographic characteristics and social determinants of health, as well as validated tools to assess psychological and social resources, and mental and physical health outcomes. The survey took approximately 15 minutes to complete.

### Demographic Characteristics and Social Determinants of Health (SDoH)

Demographic characteristics and SDoH were assessed via the following variables: age (continuous), ethnicity (non-Hispanic versus Hispanic), race (White, Black or African American, American Indian or Alaskan native, Asian or Pacific Islander, or other), marital status (single, divorced or separated, married, domestic partner, other), income (continuous) and highest level of education (less than high school, some high school, completed high school, associate’s degree, some college, completed college, beyond college). An additional social determinant, food insecurity, was operationalized via a one-item yes(1)/no(0) question: During the last 12 months, did you ever eat less than you felt you should because there wasn’t enough money to buy food? To capture the experience of adverse life events, a sum score was created from the 15-items that comprise the PRAMS questionnaire stressful life events scale (Shulman et al., 2018) which asks the following question: During the past 12 months, have any of the following things happened to you? Response options included: 1) A close family member was very sick and had to go into the hospital, 2) I got separated or divorced from my husband or partner, 3) I moved to a new address, 4) I was homeless or had to sleep outside, in a car, or in a shelter, 5) My husband or partner lost their job, 6) I lost my job even though I wanted to keep working, 7) My husband, partner, or I had a cut in work hours or pay, 8) I was apart from my husband or partner due to military deployment or extended work-related travel, 9) I argued with my husband or partner more than usual, 10) My husband or partner said they didn’t want me to be pregnant, 11) I had problems paying the rent, mortgage, or other bills, 12) My husband, partner, or I went to jail, 13) Someone very close to me had a problem with drinking or drugs, 14) Someone very close to me died, and 15) Other.

### Psychosocial Resources

Psychological Capital and Social Supports were investigated as latent variables. The Compound Psycap Scale (CPC-12) is a 12-item scale that measures self-efficacy, hope, optimism, and resilience (Lorenz et al., 2016). Responses are captured via a 6-item Likert scale (strongly disagree – strongly agree). It is a comprehensive, validated measure of psycap in the general adult population (α = .80) (Lorenz et al., 2016; Lorenz et al., 2022). The Multidimensional Scale of Perceived Social Support is a 12-item scale that measures three sources of social support (friends, family, significant other) on a 5-point Likert scale. The tool shows an internal consistency of 0.90–0.94 in a prenatal sample (Zimet et al., 1990). Mindfulness was operationalized as a measured variable using the 5-item Mindfulness Attention Awareness Scale (MAAS-5) assessed on a 6-point Likert scale. The tool shows high internal validity (α = .89–.93) (MacKillop & Anderson, 2007; Osman et al., 2016).

### Outcomes

Depression, anxiety, and chronic health conditions were investigated as outcome variables. The Patient Health Questionnaire-8 (PHQ-8) (α = 0.85) is a widely used 8-item validated diagnostic measure for depressive disorders (Kroenke et al., 2009). It shows high validity and reliability when using a cutoff score of 10. The Generalized Anxiety Disorder-7 scale is a 7-item brief measure of anxiety. When applied to a prenatal population, it shows high reliability (α = 0.89) and yielded a sensitivity of 73.3% and a specificity of 67.3% (Spitzer et al., 2006). To assess the prevalence of chronic health conditions, a one-item question asked, “Are you currently experiencing any of the following chronic health conditions?” Responses included: asthma, gestational diabetes, high blood sugar, overweight/obesity, and high blood pressure, and were assessed dichotomously (no = 0; yes = 1). A sum score was created ranging from 0 to 5.

### Data analyses

To explore the primary research question, all variables of interest were examined for missing data and multivariate outliers using missing value analysis and review of Mahalanobis Distances; 13 records were identified as outliers based on comparison to chi square distributions (values of < .001). However, results were unchanged after the exclusion of these records, so we retained all data. We investigated patterns of missing values for all variables included in our final models. Percent missingness ranged from 6.8–11.5%; however, Little’s Missing Completely At Random test (MCAR) (Little, 1988) provided evidence that data were missing complete at random (X^2^ (49, N = 340) = 49.35, p = .46) so full information maximum likelihood estimator was used to account for all available data. Demographic characteristics and SDoH including, age, race, ethnicity, education, the experience of adverse life events and food insecurity, were controlled for in the hypothesized model. Race was dichotomized (White (0), Black, Indigenous, and other People of Color (BIPOC (1)) and the categorical variables of education (high school degree or lower (0), some college or an associate degree (1), college degree or higher (2)), and marital status (single (0), married or partnered (1)) were collapsed for analyses due to small sample sizes. The number of adverse life events experienced was recoded for all analyses; a reported experience of 5 or more stressors were collapsed into one category (range = 0–5).

First, we conducted confirmatory factor analysis to assess the psychometric properties of the two latent constructs (i.e., psychological capital, social supports). Once these measurement analyses were completed, univariate distributions (means, standard deviations, and graphical displays), assumptions of normality, linearity, and homoskedasticity, and bivariate associations between all key variables were explored (Fife, 2020). We used structural equation modeling (SEM) to test hypothesized relationships simultaneously in a single model, while controlling for covariates (Kline, 2004). The model was analyzed using maximum likelihood estimation with robust standard errors in MPlus version 8.4 (Muthén & Muthén, 2017). Results were interpreted using standardized beta (β) and 95% confidence intervals (CIs), in addition to p values. Parameter estimates for path coefficients were tested for statistical significance; alpha was set at .05. Model fit was compared using the Bayesian information criterion index (BIC) among nested models to identify the best fitting model. Additional fit indices were used to determine if the hypothesized model fit well with the sample data. Specifically, root mean square error of approximation (RMSEA) < .08, and a narrow 90% confidence interval around RMSEA were indicatives of good fit (Byrne, 2013; Kenny et al., 2015). Comparative fit index (CFI) that was close to 0.95 was considered superior fit, values below 0.90 were regarded as poor fit, and Standardized Root-Mean-Square Residual (SRMR) of .08 or less were considered good fit (Byrne, 2013). Once we identified our final model, we conducted a multi-group analysis to investigate differences in the hypothesized structural model by race. Prior to multi-group structural modeling, measurement invariance was explored and confirmed to ensure that the estimated factors were measuring the same underlying latent construct within each racial group. We calculated the change in chi square between the free and constrained models to determine if the structural models were statistically significantly different between White individuals versus BIPOC.

## Results

Table 1 presents the prevalence of social determinants of health and adverse life events in the final analytical sample (n=340). Almost half of the sample (44%) reported experiencing food insecurity in the past 12 months. Twenty-six percent (26%) of the sample reported experiencing no adverse life events in the past 12-months, whereas 20% reported experiencing 1, 15% reported experiencing 2, 15% reported experiencing 3, 9% reported experiencing 4, and 15% reported experiencing 5 or more external stressors in the past 12-months. Thirty eight percent (44%) of the sample met the criteria for clinical rates of depression and 38% of the sample met the criteria for clinical rates of anxiety (both based on clinical cutoffs of 10 or greater). Approximately 1/3 of participants reported experiencing no chronic health conditions (33%), 38% reported experiencing 1, 24% reported experiencing 2, 11% reported experiencing 3, 4% reported experiencing 4, and 1% reported experiencing all 5 chronic health conditions.

Table 2 displays Pearson’s correlations between all predictor and outcome variables. Hope and optimism were significantly and negatively correlated with depression (hope: r = −.12, optimism: r = −.20, both p < .05), anxiety (hope: r = −.14, optimism: r = −.20, both p < .05), and the number of chronic health conditions (hope: r = −.14, optimism: r = −.15, both p < .05). Self-efficacy was significantly correlated with the number of chronic health conditions (r=−.12, p<.05). Overall psycap was significantly correlated with the number of chronic health conditions (r=−.13, p<.05) and mindfulness was significantly correlated with depression (r=−.27), anxiety (r=−.24), and the number of chronic health conditions (r=−.16) (all p<.01). Social support from friends, family members and significant others, as well as overall social support were significantly correlated with depression and anxiety (r values range from −.21 to −.24, all p<.01). The number of adverse life events experienced was significantly associated with all outcomes (depression: r=.34, anxiety: r=.27, chronic health conditions: r=.26, all p<.01). Depression and anxiety were strongly correlated (r=.83, p<.01); depression (r=.30) and anxiety (.40) were both moderately correlated with the number of chronic health conditions (p<.01).

**Figure 1.**
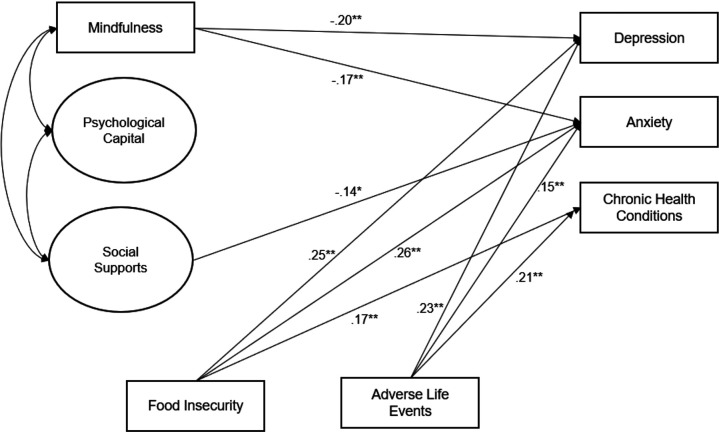
Final Single Group Structural Equation Model Fit Statistics: (χ^2^ (43) = 99.07, p<.01, CFI = 0.97, SRMR = 0.04, and RMSEA = 0. 06 (0.05 – 0.08)) *Only statistically significant paths are shown (* p<.05, **p<.01)

CFA findings confirmed the overall measurement model theory for each of the two latent variables; hope, optimism, self-efficacy and resilience loaded on the latent factor of psychological capital and friend, family, and significant other support loaded on the latent factor of social support. Fit statistics of this final two-factor model suggested good model fit (χ2 (13) = 23.71, p=.04, CFI = 0.99, SRMR = 0.03, and RMSEA = 0. 05 (.01 – 0.08)). Standardized estimates from our final structural equation model are displayed in Figure 1. To simplify the final model, only statistically significant pathways are displayed. We excluded age, education, and marital status due to a lack of associations with both exogenous and endogenous variables of interest and model fit statistics in our final model. We investigated fit indices, which suggested that the final hypothesized model had an acceptable fit with the sample data (χ2 (43) = 99.07, p<.01, CFI = 0.97, SRMR = 0.04, and RMSEA = 0. 06 (0.05 – 0.08)). Experiencing food insecurity (anx: *β* = .26, SE= .06; dep: *β* = .25, SE= .06) and higher numbers of adverse life events (anx: *β*=.15, SE= .05; dep: *β* =.23, SE= .05) were statistically significantly associated with elevated anxiety and depression scores (both p<.01). Higher levels of mindfulness was statistically significantly related to lower anxiety (*β* = −.17, SE= .06) and lower depression (*β* = −.20, SE= .05) scores (both p<.01). Higher levels of social supports were statistically significantly related to lower anxiety scores (*β* = −.14, SE= .07, p<.05), though not significantly associated with depression scores or chronic health conditions. Though psycap was moderately correlated with both mindfulness (r=.34), and social supports (r=.44), it was not independently associated with any of the outcomes (p values range from .13 to .49). No psychosocial resources were statistically significantly related to the number of chronic health conditions in the final single-group model.

Findings from the multi-group analyses are presented in Table 3. Scale measurement invariance was confirmed (χ2 (36) = 45.70, p=.13, CFI = 0.99, SRMR=0.06, and RMSEA=0. 04 (.00 – 0.07)) and the structural model was statistically significantly different between pregnant White individuals and BIPOC in this sample (Δ χ2 (27) = 116.71, p <.01). Among BIPOC, higher levels of social supports were statistically significantly related to lower anxiety (*β* = −.32, SE= .12) and lower depression (*β* = −.26, SE=.12) scores (both p<.05); however, these associations were no longer statistically significant among the White sample. Additionally, among BIPOC, higher levels of psycap were statistically significantly associated with lower numbers of chronic health conditions (*β* = −.28, SE= .13, p=.03). Though mindfulness remained a statistically significant predictor of positive mental health outcomes in both groups, parameter estimates suggest a stronger relationship among BIPOC compared to their White counterparts (BIPOC anx: *β* = −.29, SE= .11, p<.01; BIPOC dep: *β* = −.23, SE= .11, p=.03).

## Discussion

This study represents one of the first attempts to investigate associations between multi-level psychosocial resources, and physical and mental health outcomes in a sample of pregnant individuals, and how these relationships may vary by race. All psychosocial resources included in the models (i.e. mindfulness, psychological capital, and social support) were moderately correlated. In our single-group model, we found that mindfulness was statistically significant and inversely associated with prenatal anxiety and depression scores, and social supports were statistically significant and inversely associated with prenatal anxiety scores, after controlling for food insecurity and adverse life events. None of the psychosocial resources were statistically significantly associated with the number of chronic health conditions in the final single group model. Our multi-group model found that mindfulness, psychological capital, and social supports were statistically significant and inversely associated with anxiety, depression, and chronic health conditions among pregnant BIPOC; only mindfulness remained statistically significant with respect to the mental health outcomes among White individuals.

The findings from our single-group model align with past studies that suggest MBIs have a positive effect on prenatal mental health, though many studies have been conducted among high-income, predominantly White samples (Sun et al., 2022). Specifically, the use of Mindfulness-Based Cognitive Therapy (MBCT) and Mindfulness-Based Stress Reduction (MBSR) have been found to be efficacious psychosocial interventions for prenatal mental health through the targeting of mindfulness (Kuyken et al., 2008; Kuyken et al., 2010; Ma & Teasdale, 2004) (Kabat-Zinn, 1982). These prenatal MBIs may promote positive mental well-being via reductions in stress (Felder et al., 2018; Keng et al., 2011; Matvienko-Sikar et al., 2016; Shapiro et al., 2008). One potential pathway linking MBIs and reduced anxiety and depression is through adaptive coping mechanisms (Machado et al., 2021). For example, a recent integrative literature review of MBIs found that participating in mindfulness-based strategies during pregnancy led to increases in positive framing, acceptance, and instrumental and emotional support (all examples of adaptive coping techniques) (Carver, 1997). MBIs also encourage reflection, mindfulness, and awareness of the present moment, which may translate to an increased use of active coping strategies (Lavender et al., 2016). Though these coping strategies may also be related to health behaviors that contribute to decreased risk for chronic health conditions during pregnancy (e.g., mindful eating and movement (Youngwanichsetha et al., 2014)), we did not find significant associations between mindfulness and chronic health conditions in our models.

MBIs may be particularly effective at optimizing prenatal mental health, even after accounting for significant stress exposures and adverse social determinants, because of the neuroplasticity of the brain during pregnancy. Neuroplasticity is the ability of one’s mind to adapt and change as a result of stimuli through reorganization of structure and function (Puderbaugh & Emmady, 2023). Neuroplasticity is elevated during the perinatal period (Pawluski et al., 2016) to biologically allow for pregnant and postpartum individuals to adapt to their new roles and develop protective and caring maternal instincts (Barba-Müller et al., 2019). Due to this increased plasticity, neurogenesis and synaptic remodeling creates the potential for new thoughts, emotions, and habits (Roshan-Milani et al., 2021). The susceptibility of “learned helplessness” (Cabib et al., 2020), depression and anxiety, along with other stress-based mental illnesses is high, and this vulnerability to negative thoughts and behaviors is known as maladaptive neuroplasticity (Peterson, 2012). Fortunately, this increased plasticity can be also create potential for positive adaptation, and there is an opportunity for adaptive coping behaviors to be easily acquired (Cabib et al., 2020). Fostering neuroplasticity through mind-body techniques beginning during pregnancy can support a more adaptive transition and increase individuals’ ability to cope with the stressors associated with this adjustment thus promoting positive mental health and well-being.

Interestingly, though psycap was significantly correlated with both mindfulness and social supports, this psychological construct was not independently associated with any of the outcome variables in our final single-group model. This contradicts some past studies that have evaluated psycap interventions (PCIs) and found that these are evidence-based approaches that bolster psycap and positively impact numerous mental health outcomes in non-pregnant samples (Avey et al., 2009; Luthans et al., 2007; Luthans & Youssef-Morgan, 2017; Youssef-Morgan & Luthans, 2015). Specifically, PCIs have been found to increase job satisfaction, job engagement, mental health and well-being and decrease stress and substance use (Newman et al., 2014; Rabenu et al., 2017). However, PCIs have primarily been tested in organizational settings with employee and student populations (Dello Russo & Stoykova, 2015; Lupşa et al., 2020; Luthans et al., 2008). No studies have investigated associations between psycap and prenatal mental health or adapted PCIs specifically for prenatal populations. A possible hypothesis that warrants future investigation is that mindfulness may be more efficacious in mitigating negative perinatal health outcomes such as anxiety(Shi & MacBeth, 2017), whereas psycap may be a stronger predictor of flourishing and positive health outcomes (e.g. well-being, work and life satisfaction)(Youssef-Morgan & Luthans, 2015). Though our findings suggest that psycap may support multi-level resource acquisition and thus help to mitigate a cascade of personal and/or social losses that often occur in the perinatal period, MBIs may be more efficacious with respect to mitigating prenatal depression and anxiety compared to PCIs.

In this sample, social resources were found to decrease the risk of experiencing prenatal anxiety. These findings align with extensive literature that demonstrates the beneficial influence of social support on prenatal mental health and neonatal outcomes (Zhou et al., 2017). A recent study of 2,341 pregnant individuals found that lacking social support, particularly from partners/significant others, was associated with elevated depressive symptoms and that these individuals were also less likely to access prenatal care (Sidebottom et al., 2017). A systematic review of 64,449 pregnant individuals found a significant relationship between low social support and prenatal mood disorders, including both depression and anxiety (Bedaso et al., 2021). A final study measured reassurance of worth and reliable alliance, which are two aspects of social support, and found that they were strongly correlated with both depression and anxiety in pregnancy (Milgrom et al., 2019).

Multi-group analyses suggest that psychosocial resource interventions that target mindfulness, psychological capital and various sources of social support may be particularly protective for pregnant BIPOC and be associated with better mental and physical health outcomes. A meta-analysis of 17 studies examining prenatal MBIs found significant improvements in depressive symptoms (Lever Taylor et al., 2016). However, effects were generally small-to-moderate, often treatment-oriented rather than prevention-oriented (Nillni et al., 2018), and few interventions are targeted specifically towards promoting multi-level resources (Matvienko-Sikar et al., 2016; Sin & Lyubomirsky, 2009). Very few MBIs have studied the impact on both mental and physical health outcomes during pregnancy. Though insufficient studies exist related to the implementation of MBIs and psychosocial interventions among low-resourced individuals, a systematic review investigated 24 RCTs that were implemented with low-income individuals and found that MBIs resulted in a small but statistically significant improvement in mental health and well-being outcomes compared to controls. An alternative study implemented an MBI among high-risk pregnant individuals experiencing external stressors and multiple physical and mental health comorbidities found that the program significantly decreased anxiety levels (Waldron et al., 2018). The relationships between social support and prenatal mood disorders in racial and ethnic minority individuals is also unclear. A large recent study that sought to investigate these relationships found that higher levels of social support decreased the risk for experiencing perinatal depression and that these effects did not differ by race or ethnicity (Pao et al., 2019). However, an alternative study found that MBIs that had a higher proportion of BIPOC had larger effect sizes which aligns with the findings in the current study (Sun et al., 2022). Similarly, we found that social support may be associated with a decreased risk of experiencing both depression and anxiety specifically among pregnant BIPOC, thus highlighting the need for future work to investigate additional types (e.g., instrumental, emotional) and sources (e.g., family, friend, significant other, healthcare provider) of social resources that are most protective among pregnant people of color (Pao et al., 2019). Finally, within the BIPOC subsample, higher levels of psycap were associated with lower numbers of chronic health conditions. Additional work is needed for further investigate this relationship but psycap, and self-efficacy, optimism and hope in particular, may be positively associated with health literacy and the adoption of healthy behaviors translating to decreased risk for chronic health conditions (O’Leary, 1985; Schiavon et al., 2017).

Rigorous cultural adaptation of current psychosocial interventions to address the needs of minoritized individuals is paramount since most of these interventions have been tested in middle- to high-income, White samples. This necessitates the need for qualitative research to better understand how to increase engagement, participation and adherence, as well as adaptation frameworks to ensure interventions are adapted and implemented using community-centered approaches (Hwang, 2009). Adaptations should account for the inherent strengths and lived experiences of stress, the comorbidity of mental and physical health outcomes, and the multi-level resources that are needed to decrease structural-related gaps in prevention and treatment programs (Crane et al., 2017). A past study suggests that the inclusion of culturally-relevant and culturally-validated instruments as outcome measures (e.g., acculturation, mistrust, trauma, discrimination) may further inform the mechanisms linking psychosocial resource interventions and well-being outcomes among individuals experiencing discrimination (Sun et al., 2022).

Our findings reinforce the need for psychosocial interventions that target multi-level resources. This is echoed by a recent study that suggest individuals experiencing significant structural demands may benefit from a multi-level intervention that addresses individual- interpersonal- and community-level factors affecting physical and mental health outcomes (Sun et al., 2022). Because racism-related stress during pregnancy is associated with significant mental health costs and maternal morbidity and mortality outcomes, mindfulness and valued living (MVL)-based strategies may be protective by targeting stress appraisals, specifically related to the experience of discrimination. For example, a recent study suggests that MVL-based strategies for people of color may result in the acquisition of new psychological resources including self-compassion, coping, flexibility, and engagement in values-based actions which may increase individuals resource reservoirs during pregnancy and protect again poor perinatal mental health outcomes (Martinez et al., 2022).

A recent meta-analysis of prenatal psychosocial interventions highlighted the need for more research to establish when, which, how and for whom these interventions can be suitable (Corno et al., 2019). In further support of this need, the World Health Organization (WHO) has recently emphasized the priority of expanding the concept of health and embracing a perspective that maximizes population mental health and well-being (WHO, 2018). Specifically, the WHO coined the concept of a ‘positive pregnancy experience’ which includes not only the treatment of diseases, but also prevention and well-being promotion. Theoretically and practically, there is a gap in our knowledge regarding identification and understanding of how multi-level protective factors may reduce mental and physical illness and optimize well-being among low-resourced and minority communities.

Though this study has significant strengths, it is not without limitations. The sample is not representative of all pregnant individuals in the United States due to the convenience sampling approach using for recruitment. Panel recruitment also may impact the transparency of the data and could present challenges related to data quality. However, our findings confirm much of the past literature that investigated singular resources (e.g., mindfulness) and associations with prenatal mental health outcomes. Additionally, these data are cross-sectional, thus limiting our interpretation of causality.

## Future Research

Individuals who acquire and maintain a resource reservoir may be more likely to utilize adaptive coping mechanisms to combat stress, thus exhibiting resistance to disadvantage and resulting in positive health outcomes (Gallo et al., 2009; Hobfoll, 2002, 2011). As resources travel in caravans and collectively impact mental health and well-being, our findings confirm studies that suggest a “shotgun” approach in which individuals practice cultivating several multi-level resources across the prenatal period may be more effective than focusing on one particular resource (Phan et al., 2020; Sin & Lyubomirsky, 2009). Future work should further investigate promising psychosocial resources, such as gratitude, cognitive and structural social capital, and neighborhood attachment, that may further promote positive mental and physical health outcomes and overall well-being during pregnancy. Moreover, past literature suggests mixed findings regarding the acceptability of current MBIs among the BIPOC community (Sun et al., 2022). Identification of core components of these interventions, consideration of upstream structural determinants and MVL-based strategies, cultural adaptation, and an emphasis on resilience rather than psychopathology may result in larger effect sizes and improved prenatal mental and physical health outcomes as well as overall well-being among pregnant individuals traditionally underrepresented in research.

## Data Availability

Data is available from the authors upon request.
